# Perceived Quality of Traditional Chinese Medicine Care in Community Health Services: A Cross-Sectional Survey in Hangzhou of China

**DOI:** 10.1155/2022/7512581

**Published:** 2022-07-11

**Authors:** Xinyu Zhang, Jianping Ren, Liqi Sun, Chaojie Liu

**Affiliations:** ^1^School of Public Health, Hangzhou Normal University, Hangzhou 311121, China; ^2^School of Humanities and Health Management, Jinzhou Medical University, Jinzhou 121001, China; ^3^Gusu District Health Supervision Office, Suzhou 215000, China; ^4^School of Psychology and Public Health, La Trobe University, Melbourne 3086, VIC, Australia

## Abstract

**Background:**

Traditional Chinese medicine (TCM) is an integral part of the mainstream health care system in China. Public community health services are required by the government to deliver TCM services. This study aimed to assess patient perceived quality of TCM care in community health services.

**Methods:**

A cross-sectional questionnaire survey was conducted on 471 TCM users in four community health centers in Hangzhou. Respondents were asked to rate their experiences on a Likert scale about tangibility, reliability, responsiveness, assurance and empathy of the TCM services they received. Linear regression models were established to determine the sociodemographic and services factors associated with the ratings.

**Results:**

Average ratings on the five aspects of the TCM care ranged from 78 to 88 out of a possible 100, with assurance attracting the highest and empathy attracting the lowest score. Overall, higher perceived quality of TCM care (except for assurance) was associated with a choice of TCM in preference to western medicine. Those who reported higher cost (≥100 yuan) of TCM care rated higher on responsiveness and empathy of the care. But higher frequency of visits to community TCM services was associated with lower ratings on reliability, assurance and empathy. Those who received two or more TCM modalities also perceived lower tangible care. In addition, higher ratings on reliability and responsiveness were found in women. The respondents with a university qualification gave higher ratings on reliability and responsiveness; by contrast, those with a highest education of senior high school rated lower on assurance and empathy. Lower perceived tangibility and assurance was also associated with rural residency. Compared with those working in the public sector, the respondents from the retail and services sector gave a higher rating on assurance but a lower rating on empathy.

**Conclusion:**

Overall, the TCM users perceived high quality of TCM care in community health services in Hangzhou. However, there is a need to further improve TCM care from all quality perspectives in order to attract and maintain consumer trust in TCM.

## 1. Background

Traditional Chinese Medicine (TCM) is one of the most widely used forms of complementary and alternative medicine (CAM). In 2002, the World Health Organization (WHO) launched “Traditional Medicine Strategy 2002–2005” for member States to guide safe and effective use of CAM [1]. Its most recent update “Traditional Medicine Strategy 2014–2023” attempts to promote a global integration of CAM into mainstream health systems [2]. Empirical evidence shows that CAM is becoming increasingly used in developed countries. In the United States, for example, 59 million people tried a CAM therapy and spent $30.2 billion on CAM in 2012. In many low- and middle-income countries, CAM may be the only form of care that is affordable, available and accessible. The WHO estimated that up to 80% of African populations rely, either partly or completely, on traditional medicines for their basic healthcare needs [3]. Many developing countries have attempted to integrate traditional medicine into their primary care systems [3–5].

TCM care involves a range of modalities such as acupuncture, moxibustion, herbal medicine and therapeutic massage which have spread over the world widely. In the Tuscan Network of Integrative Medicine, for example, more than 75 public services offer acupuncture and herbal medicine [4]. TCM has played a pivotal role in the health system development in mainland China since the People's Republic of China was established [6]. It has been incorporated into the entire spectrum of health care services, ranging from prevention of diseases to acute care in hospitals, long term care and rehabilitation [7]. However, the development of TCM was jeopardized as China adopted a market-driven approach in health reform in the 1980s and 1990s when allopathic medicine and hospital care prevailed [8]. Since then, health care services have attracted increasing criticisms for their expensive and fragmented approaches. To revitalize TCM, the central government announced a policy in 2006, requiring all community health centers to have at least one doctor specializing in TCM [9]. It was estimated that, by 2009, 51.6% of community health centers [10] and 22% of medical practitioners [11] had provided TCM services. In 2016, the central government re-emphasized the importance of TCM care and set up a goal for universal coverage of TCM in community health services by 2020 [12].

TCM services are delivered in different ways under different systems. In mainland China, TCM has been promoted as a major strategy for universal access to primary care. This includes development of one TCM hospital for each county and wide availability of TCM services in non-TCM health institutions [13]. According to the National Administration of TCM, China established 43 TCM universities/colleges. In 2018, these universities employed more than 48,300 faculty members and enrolled over 729,000 students [14]. TCM practitioners are registered as medical doctors in parallel with their counterparts specialized in western medicine under the same legislation framework. The Chinese government also planned to establish 30 overseas centers by the end of 2020 [15]. The TCM services system in mainland China is characterized with a dominance of public provision, large institutions, and strong integration between TCM and western medicine. These features are quite different from those in Hong Kong, Taiwan and other regions where TCM services are also widely available despite a consistent approach in modernization of TCM workforce development through tertiary education [16]. It is important to note that TCM services are covered by social health insurance programs in mainland China [17], which involve more than 1300 TCM products and 892 components [18]. TCM products have also been used for preventing infections and treating patients during the outbreaks of severe acute respiratory syndrome (SARS) and novel coronavirus pneumonia (COVID-19) [19].

It is evident that TCM care in community health services is increasing rapidly in mainland China with the mandate from the government. In Hangzhou, where this study was conducted, TCM visits in community health services increased by 47.11% from 2012 to 2015. The percentage of TCM visits accounted for 25.46%, 26.87%, and 30.64% of all visits to community health services in 2013, 2014 and 2015, respectively [20]. However, it is not clear whether the quality of TCM care is also well accepted by its users. This study aimed to answer this question through a cross-sectional survey on TCM users in community health services in Hangzhou. Efforts to improve the quality of CAM services have been strongly advocated by the WHO [2]. Findings of this study will not only provide evidence support for better policy development regarding TCM services in primary care in China, but also advance our understanding of the needs of TCM users in general.

## 2. Methods

This study adopted a cross-sectional design. The study was undertaken in Hangzhou, one of the most developed municipalities in China with over 9.8 million permanent residents. Hangzhou is divided into 13 local jurisdictions and the majority (over 77%) of its residents live in the 10 urban districts. In 2019, its per capita GDP reached 152,000 yuan (US$22,969), much higher than the national average of 71,000 yuan (US$10,729). Hangzhou established 129 community health centers, 100 of which have a dedicated TCM unit. The total volume of TCM visits in community health services have exceeded 10 million since 2013 [21].

Ethics approval for the study protocol was obtained from Hangzhou Normal University (Reference number 20190070). The survey was anonymous and verbal informed consent was obtained prior to proceeding of the survey.

### 2.1. Sampling

Participants of this study were selected using a multi-stage sampling strategy. Four urban districts (Shangcheng, Xiacheng, Jianggan and Gongshu) were purposively identified first, representing different levels of economic development and geographical locations in Hangzhou. Per capita GDP of the four districts ranged from 12,218 USD (Jianggan) to 42,728 USD (Shangcheng) in 2017 [22]. In each district, an average-sized community health center with a well-established TCM unit was selected.

About 3,200 patients visited the selected TCM units over the period of the survey (1–4 July 2017) and 500 adult patients (≥18 years) were conveniently approached by the trained data collectors to participate in the survey. Of those invited, 471 (94.2%) completed the survey. This sample size allowed us to make a reliable estimation of the quality ratings and perform linear regression modelling on the ratings with up to 50 independent variables [23].

### 2.2. Instruments

Data were collected using a self-developed questionnaire. The questionnaire comprised two sections. The first section investigated the participants' use of health care services based on Andersen's behavioral model [24]. It captured needs factors (measured by the demographic characteristics of respondents, chronic conditions, and a self-rating on overall health) and enabling factors (measured by marital status, education, income, job, residency, and health insurance). Previous studies show that these variables are significant predictors of health and health care outcomes [25, 26]. In this study, chronic condition was identified from a list of diagnosed conditions, including hypertension, diabetes, gout, cardiovascular and cerebrovascular diseases, digestive diseases, chronic obstructive pulmonary disease, liver disease, kidney disease, and tumor. Self-rating on overall health was assessed on a five-point Likert scale, which was then recoded into three categories (good, fair and poor) in data analyses. Income was estimated as monthly household average income per capita. Residency was defined by the household registration system “Hukou.” In China, welfare entitlements are attached to local Hukou registrations. China has established almost universal health insurance coverage thanks to multiple Hukou-based funds subsidized by the government [26]. These funds can be categorized into three types: basic health insurance for urban employees, basic health insurance for urban residents, and new rural cooperative medical scheme. Overall, urban employees enjoy a higher level of entitlements than others.

The questionnaire also captured the frequency, type, and cost of TCM care services, which encompassed medicine conditioning, acupuncture, massage, cupping, scraping, fumigation, acupoint injection, moxibustion, “hot ironing,” and traditional treatment for bone injuries.

The second section assessed patient perceived quality of the current TCM visit in community health services using the SERVQUAL framework proposed by Parasuraman and colleagues [27–29]. It is perhaps the most commonly used framework for measuring quality of healthcare services in both developed and developing countries [30]. The SERVQUAL framework taps into five dimensions of quality of care: tangibility, reliability, responsiveness, assurance and empathy. Tangibility measures accessibility to physical and human resources. Reliability indicates the ability to accurately and reliably complete the promised services. Responsiveness captures the adequacy of service providers to meet consumer requests. Assurance reflects trust and confidence of consumers on the competency of service providers. Empathy addresses personalized needs and context [31]. Minor modifications were made on the SERVQUAL instrument after two rounds of consultations with 15 experts and interviews with 20 TCM users for the purpose of adaptation to the context of TCM services in China. For example, responsiveness involved simplification of services procedures and disclosure of information about the practitioners. This resulted in an adapted version of SERVQUAL, comprising 23 items, with an overall Cronbach's *α* coefficient of 0.936, well above 0.70 as required [32]. The final data analyses further excluded three items since deletion of these items produced a higher Cronbach's *α* coefficient for their respective domains in the pilot study involving 100 participants. Three items were “Q12 The institution provides convenience services such as consultation, consultation, and triage,” “Q17 The number of Chinese medicine personnel and the allocation of professional titles are reasonable, which can meet your medical needs,” “Q21 Doctors provide you with personalized service.” The finalized SERVQUAL-based community TCM health service evaluation questionnaire is shown in the (available (here)) appendix. The exploratory factor analysis (varimax rotation) with the final sample (*n* = 471) suggested a five-factor structure of the instrument, supporting the construct validity of the instrument confirmed by the studies in Asian populations including in China [33, 34].

### 2.3. Data Collection

The questionnaire was administered through face-to-face interviews in the participating community health centers. Eight interviewers were trained over a two-day workshop. They were taught about how to follow the protocol, how to initiate a conversation with the study participants appropriately considering their literacy level, and how to avoid bias and ensure completeness of data.

The trained interviewers were paired and deployed to the selected community health centers. However, they worked independently. Data were collected at the customer services area. Patients who had completed the TCM care were approached whenever one of the interviewers was available. On average, each interviewer collected 15 questionnaires per day. Each interview took about 13 minutes (ranging from 10 to 20 minutes).

The interviewers explained the purpose and procedure of the study and obtained oral informed consent from the participants prior to the survey. Participation in the survey was completely voluntary. The interviewers had no servicing relationships with the interviewees.

### 2.4. Data Analysis

The primary outcome of this study was perceived quality of the TCM care reflected on five domains: tangibility, reliability, responsiveness, assurance and empathy. Each quality item in the questionnaire was rated on a five-point Likert scale, with a higher score indicating a higher level of quality of care. A summed score was then calculated for each quality domain and subsequently transformed into a score ranging from 0 to 100 [35]. The score was interpreted as a continuous quality spectrum. Means and standard deviations of quality scores were presented.

The secondary outcome of this study examined variations of perceived quality of the TCM care and determinants of the variations. Student's *t*-tests or analysis of variance (*F* tests) for independent samples were performed to examine the statistical differences in quality scores across groups of respondents with different characteristics. Multivariate linear regression models were established to identify the independent variables associated with the five domains of quality ratings. A stepwise approach was adopted in the modelling involving all the tested independent variables (section one of the questionnaire). Missing data, if any, were handled through listwise deletion.

Data were double entered into EpiData 3.1 to ensure accuracy. Statistical analyses were performed using SPSS 21.0. A *p* value at 0.05 (two sides) was set for statistical significance.

## 3. Results

### 3.1. Characteristics of Respondents

The majority (67.7%) of respondents were women; 66.5% were older than 45 years and 83.7 were married at the time of the survey. Although most respondents had a local household registration, 17.8% did not. Correspondingly, the respondents were predominantly covered by the two urban insurance programs. The distribution of respondents was roughly even across different levels of education. More than 42% of respondents had a monthly household income of less than 5,000 Yuan per capita, compared with an average salary of 5,389 yuan in Hangzhou in 2018. About 17% of respondents rated their health as poor and 36.5% reported one or more chronic conditions. Only 32% of respondents resided within 15 minutes of walking distance (a government target) to the nearest community health facility. Secondary hospitals were the least preferred healthcare provider. Although only 18.7% of respondents chose TCM as preferred care explicitly, 41.2% preferred integrated TCM and western medicine. About 28.5% of respondents used TCM services in the community health centers for the first time. Over 64% received two or more TCM modalities, but predominantly (90.2%) at a cost lower than 100 yuan (US$15) (Table 1).

### 3.2. Perceived Quality of TCM Care

The respondents gave an average rating of 82.52 (SD = 12.05) for tangibility, 83.14 (SD = 10.96) for reliability, 79.63 (SD = 11.77) for responsiveness, 87.64 (SD = 11.84) for assurance, and 78.27 (SD = 13.12) for empathy. The ratings varied by gender (reliability), education (reliability, responsiveness and empathy), job (empathy), health insurance (tangibility and assurance), chronic conditions (empathy), preferred health providers (assurance and empathy), frequency of visits to community health services (reliability, responsiveness and assurance), TCM modalities (tangibility and responsiveness), and TCM cost (reliability, responsiveness and empathy). Preferred care, first time visits, and frequency of TCM care received in community health services were associated with variations in ratings on all of the five dimensions of quality of care (Table 2).

The multivariate linear regression models confirmed that gender, education, job, health insurance, preferred care, frequency of TCM care received in community health services, TCM modalities and care cost were significant predictors of quality ratings after adjustment for variations in other variables (Table 3). Overall, higher perceived quality of TCM care (except for assurance) was associated with a choice of TCM in preference to western medicine. Those who reported higher cost (≥100 yuan) of TCM care rated higher on responsiveness and empathy of the care. But higher frequency of visits to community TCM services was associated with lower ratings on reliability, assurance and empathy. Those who received two or more TCM modalities also perceived lower tangible care. In addition, higher ratings on reliability and responsiveness were found in women.

The respondents with a university qualification gave higher ratings on reliability and responsiveness; by contrast, those with a highest education of senior high school rated lower on assurance and empathy. Lower perceived tangibility and assurance was also associated with rural residency. Compared with those working in the public sector, the respondents from the retail and services sector gave a higher rating on assurance but a lower rating on empathy.

## 4. Discussion

Overall, respondents of this study reported high levels of quality of TCM care in community health services, with average ratings ranging from 76 to 88 out of a possible 100 across the five dimensions of quality. Similar to some other studies [36, 37], assurance attracted the highest rating in this study, compared with the lowest rating on empathy. In recent years, the Chinese government has attached great importance to the development of TCM in community health services by introducing a series of policies and investment. The relatively higher rating on assurance may simply reflect the growing capability and competency of the TCM workforce. But the relatively lower rating on empathy indicates that the strength of holistic and personalized approach in TCM may not have been fully functioning. This study showed that patient ratings on quality of TCM care have no correlation with their health needs as measured by the modern concept of disease and health. But modernized health facilities, including community health services in China, are often designed around the needs of health providers, instead of consumers [38]. TCM may deviate from its tradition when it is integrated into the mainstream environment dominated by allopathic medicine [39]. TCM services can therefore suffer, becoming increasingly crowded, fragmented and episodic [40].

Studies in some other countries revealed that CAM users are more likely to be women and well educated [41, 42]. In this study, we found that female TCM users in community health services rated higher on reliability and responsiveness of TCM services than their male counterparts, which is consistent with findings of studies conducted elsewhere [43]. But there is not a consistent pattern in the associations between education and perceived quality of TCM care. We found that those with a university qualification rated higher in reliability and responsibility, which is consistent with Zun's findings [44]. This appears to be contradictory with potential higher expectations held by this group of users [45]. However, we also found that the TCM users who completed senior high school rated lower in assurance and empathy compared with their less educated counterparts.

The finding of higher assurance rating and lower empathy rating of retail and services workers is interesting. It highlights the importance of quality assessment from multiple perspectives. Internationally, retail and services workers tend to hold lower qualifications, which can jeopardize their chance of getting personalized needs met [46].

Urban-rural disparities in perceived tangibility and assurance of TCM care deserves further investigations. Urban-rural inequalities in health care and health outcomes have been a major policy concern in China [6]. This study was conducted in urban community health settings. Rural respondents are likely to feel less engaged than their urban counterparts [47].

Trust is a strong enabler of TCM use [48]. Indeed, a choice of western medicine in preference to TCM was found in this study to be a significant predictor of lower quality ratings on TCM care. Higher quality ratings of TCM care were also found to be associated higher spending on TCM. It is important to note that the price of TCM care is overwhelmingly low in China [49]. The relatively higher spending is perhaps an indicator of higher willingness to accept TCM care.

It is a great challenge to maintain trust. This study found lower ratings on assurance and empathy in those who most frequently received TCM care (≥10) in community health services. The results are consistent with the findings of a study conducted elsewhere [44]. Accumulated visits may increase the expectation of consumers, leading to deflated ratings on quality of care [50]. We also found that receiving two or more TCM modalities is associated with lower ratings on tangibility. Health consumers nowadays hold very high expectations on modern technologies. TCM care usually requires long term compliance. Adding up more TCM modalities may not help but jeopardizing the confidence of consumers [51]. A study in Hong Kong showed that a belief of TCM efficacy is not enough to translate into preferred care [48]. Consumer trust in TCM needs to be strengthened through its whole-person approach and high levels of empathy. Unfortunately, empathy attracted the lowest score among the five dimensions of quality assessed in this study.

This study has several limitations. Firstly, it adopted a cross-sectional design and no causal inferences can be drawn. The study did not investigate how and why respondents chose TCM care in community health services. Secondly, the quality ratings on TCM care may be biased by its users. The study was conducted in Hangzhou, one of the most developed regions in China. The study sample is not representative of China. In addition, the study was conducted in urban settings. Rural residents are under-represented. Given the large urban-rural differences, further studies are needed to examine the views of rural TCM users in rural settings. A study conducted in Singapore shows that low-income residents are more likely to choose community CAM services than their richer counterparts [50]. Thirdly, the SERVQUAL instrument does not measure service outcomes. As a result, we are currently exploring the use of Goal Attainment Scale to measure TCM service outcomes, which are highly personalized [52]^.^

## 5. Conclusions

Overall, the quality of TCM care is well recognized by its users in community health services in Hangzhou, in particular among women and those who have a choice of TCM in preference to western medicine. Enhancing TCM care can bring benefits to the growth of community health services. However, there is a need to further improve TCM care from all quality perspectives in order to attract and maintain consumer trust in TCM.

## Figures and Tables

**Table 1:** Characteristics of respondents and TCM services.

Characteristics		Number (%)^*∗*^ of respondents				
		Shangcheng	Xiacheng	Gongshu	Jianggan	Total
Gender	Male	32 (30.5)	39 (31.5)	42 (37.8)	39 (29.8)	152 (32.3)
	Female	73 (69.5)	85 (68.5)	69 (62.2)	92 (70.2)	319 (67.7)

Age (years)	18–25	0 (0.0)	11 (8.9)	6 (5.4)	3 (2.3)	20 (4.2)
	26–45	7 (6.7)	56 (45.2)	47 (42.3)	28 (21.4)	138 (29.3)
	46–65	60 (57.1)	51 (41.1)	41 (36.9)	73 (55.7)	225 (47.8)
	>65	38 (36.2)	6 (4.8)	17 (15.3)	27 (20.6)	88 (18.7)

Residency	Local	94 (89.5)	83 (66.9)	97 (87.4)	113 (86.3)	387 (82.2)
	Non-local	11 (10.5)	41 (33.1)	14 (12.6)	18 (13.7)	84 (17.8)

Education	≤ Primary school	32 (30.5)	11 (8.9)	15 (13.5)	41 (31.3)	99 (21.0)
	Junior high school	32 (30.5)	28 (22.6)	24 (21.6)	32 (24.4)	116 (24.6)
	Senior high school	21 (20.0)	34 (27.4)	31 (27.9)	34 (26.0)	120 (25.5)
	University	20 (19.0)	51 (41.1)	41 (36.9)	24 (18.3)	136 (28.9)

Marital status	Single	1 (1.0)	23 (18.5)	15 (13.5)	5 (3.8)	44 (9.3)
	Married	93 (88.6)	97 (78.2)	86 (77.5)	118 (90.1)	394 (83.7)
	Divorced/Widowed	11 (10.5)	4 (3.2)	10 (9.0)	8 (6.1)	33 (7.0)

Monthly householdincome per capita (¥)	<5000	61 (58.1)	38 (30.6)	37 (33.3)	64 (48.9)	200 (42.5)
	5000–9999	36 (34.3)	50 (40.3)	62 (55.9)	55 (42.0)	203 (43.1)
	≥10000	8 (7.6)	36 (29.0)	12 (10.8)	12 (9.2)	68 (14.4)

Job	Public institution	5 (4.8)	13 (10.5)	12 (10.8)	10 (7.6)	40 (8.5)
	Corporate company	6 (5.7)	36 (29.0)	26 (23.4)	15 (11.5)	83 (17.6)
	Retail and services	9 (8.6)	19 (15.3)	20 (18.0)	13 (9.9)	61 (13.0)
	Retired	72 (68.6)	26 (21.0)	36 (32.4)	66 (50.4)	200 (42.5)
	Self-employed	10 (9.5)	19 (15.3)	10 (9.0)	21 (16.0)	60 (12.7)
	Others	3 (2.9)	11 (8.9)	7 (6.3)	6 (4.6)	27 (5.7)

Health insurance	Urban employee	74 (70.5)	86 (69.4)	79 (71.2)	100 (76.3)	339(72.0)
	Urban residents	22 (21.0)	26 (21.0)	29 (26.1)	20(15.3)	97 (20.6)
	Rural residents	9 (8.6)	12 (9.7)	3 (2.7)	11 (8.4)	35 (7.4)

Chronic condition	Yes	57 (54.3)	29 (23.4)	37 (33.3)	49 (37.4)	172 (36.5)
	No	48 (45.7)	95 (76.6)	74 (66.7)	82 (62.6)	299 (63.5)

Perceived health	Poor	20 (19.0)	17 (13.7)	13 (11.7)	30 (22.9)	80 (17.0)
	Fair	41 (39.0)	68 (54.8)	62 (55.9)	74 (56.5)	245 (52.0)
	Good	44 (41.9)	39 (31.5)	36 (32.4)	27 (20.6)	146 (31.0)

Distance to nearest communityhealth center (minutes)	≤15	36 (34.3)	38 (30.6)	36 (32.4)	41 (31.3)	151 (32.1)
	16–30	30 (28.6)	38 (30.6)	44 (39.6)	47 (35.9)	159 (33.8)
	>30	39 (37.1)	48 (38.7)	31 (27.9)	43 (32.8)	161 (34.2)

Preferred health provider	Community facility	67 (63.8)	78 (62.9)	90 (81.1)	97 (74.0)	332 (70.5)
	Secondary hospital	10 (9.5)	12 (9.7)	0 (0.0)	12 (9.2)	34 (7.2)
	Tertiary hospital	28 (26.7)	34 (27.4)	21 (18.9)	22 (16.8)	105 (22.3)

Preferred health care	TCM	17 (16.2)	26 (21.0)	8 (7.2)	37 (28.2)	88 (18.7)
	Western medicine	36 (34.3)	49 (39.5)	50 (45.0)	54 (41.2)	189 (40.1)
	Integrated	52 (49.5)	49 (39.5)	53 (47.7)	40 (30.5)	194 (41.2)

First visit to the TCM unit	Yes	28 (26.7)	37 (29.8)	36 (32.4)	33 (25.2)	134 (28.5)
	No	77 (73.3)	87 (70.2)	75 (67.6)	98 (74.8)	337 (71.5)

Visits to community healthinstitutions over the past month	<5	23 (21.9)	71 (57.3)	48 (43.2)	39 (29.8)	181 (38.4)
	5–9	67 (63.8)	50 (40.3)	60 (54.1)	55 (42.0)	232 (49.3)
	≥10	15 (14.3)	3 (2.4)	3 (2.7)	37 (28.2)	58 (12.3)

Visits to community TCMover the past month	<5	27 (25.7)	74 (59.7)	54 (48.6)	42 (32.1)	197 (41.8)
	5–9	63 (60.0)	48 (38.7)	54 (48.6)	55 (42.0)	220 (46.7)
	≥10	15 (14.3)	2 (1.6)	3 (2.7)	34 (26.0)	54 (11.5)

Average TCM cost per visit (¥)	<50	28 (26.7)	41 (33.1)	26 (23.4)	47 (35.9)	142 (30.1)
	50–99	68 (64.8)	59 (47.6)	82 (73.9)	74 (56.5)	283 (60.1)
	≥100	9 (8.6)	24 (19.4)	3 (2.7)	10 (7.6)	46 (9.8)

TCM modalities received inthe current visit	<2	17 (16.2)	42 (33.9)	36 (32.4)	73 (55.7)	168 (35.7)
	2	25 (23.8)	37 (29.8)	36 (32.4)	26 (19.8)	124 (26.3)
	>2	63 (60.0)	45 (36.3)	39 (35.1)	32 (24.4)	179 (38.0)

Purpose of the current visit	Disease treatment	68 (64.8)	64 (51.6)	56 (50.5)	77 (58.8)	265 (56.3)
	Preventive care	9 (8.6)	35 (28.2)	35 (31.5)	14 (10.7)	93 (19.7)
	Rehabilitation	28 (26.7)	25 (20.2)	20 (18.0)	40 (30.5)	113 (24.0)

*Note*. ^*∗*^Missing values were not included in the statistics; TCM–traditional Chinese medicine.

**Table 2:** Quality ratings (Mean ± SD) on TCM care by characteristics of respondents.

Characteristics of respondents	Tangibility	Reliability	Responsiveness	Assurance	Empathy
Gender					
Male	81.41 ± 12.01	81.50 ± 12.01	78.46 ± 11.43	87.63 ± 13.21	75.99 ± 14.29
Female	83.14 ± 12.06	83.97 ± 10.37	80.56 ± 12.16	89.30 ± 11.20	77.36 ± 14.69
Group comparison (*p*)	0.154	0.022	0.074	0.153	0.340

Age (years)					
18–25	84.40 ± 14.15	84.00 ± 14.57	83.00 ± 15.78	86.75 ± 15.50	79.67 ± 17.64
26–45	82.52 ± 12.08	83.65 ± 11.70	80.54 ± 12.14	87.78 ± 12.75	75.65 ± 14.99
46–65	82.98 ± 12.06	83.08 ± 10.69	79.84 ± 11.92	89.07 ± 11.30	76.92 ± 14.94
>65	80.91 ± 11.52	82.27 ± 9.57	78.01 ± 10.55	89.92 ± 10.98	78.51 ± 11.16
Group comparison (*p*)	0.498	0.808	0.336	0.882	0.817

Residency					
Local	82.82 ± 12.16	83.27 ± 11.04	79.99 ± 11.72	89.16 ± 11.91	77.51 ± 14.65
Non-local	81.33 ± 11.61	82.70 ± 10.75	79.40 ± 11.77	86.90 ± 11.77	74.17 ± 13.90
Group comparison (*p*)	0.306	0.664	0.686	0.116	0.056

Education					
≤ Primary school	82.46 ± 12.71	81.78 ± 10.26	78.59 ± 11.35	87.88 ± 12.27	78.65 ± 12.27
Junior high school	82.97 ± 12.07	84.62 ± 10.30	79.42 ± 11.01	88.42 ± 10.08	79.17 ± 12.62
Senior high school	80.93 ± 10.73	80.44 ± 11.04	77.13 ± 11.49	85.33 ± 12.67	74.28 ± 12.23
University	83.69 ± 12.64	85.34 ± 11.31	82.94 ± 12.40	88.93 ± 11.994	80.91 ± 14.25
Group comparison (*p*)	0.318	0.001	0.001	0.081	0.001

Monthly household income per capita (¥)					
<5000	83.24 ± 13.29	82.85 ± 11.65	79.20 ± 12.23	86.35 ± 12.60	79.43 ± 13.30
5000–9999	82.63 ± 11.52	82.66 ± 10.53	79.47 ± 11.21	88.55 ± 11.08	77.03 ± 13.08
≥10000	83.25 ± 9.76	85.60 ± 10.04	81.68 ± 12.14	88.91 ± 11.50	78.89 ± 12.74
*Group comparison* (*p*)	0.831	0.135	0.304	0.113	0.172

Marital status					
Single	83.73 ± 13.44	84.81 ± 13.00	82.04 ± 13.72	87.44 ± 13.43	78.22 ± 15.00
Married	82.69 ± 11.77	82.93 ± 10.79	79.26 ± 11.50	87.42 ± 11.69	78.38 ± 13.13
Divorced/Widowed	79.39 ± 13.42	83.84 ± 10.38	81.45 ± 12.28	90.91 ± 11.14	77.78 ± 10.76
Group comparison (*p*)	0.254	0.516	0.217	0.265	0.968

Jobs					
Public institution	86.60 ± 13.24	87.08 ± 12.18	84.20 ± 11.63	89.25 ± 11.85	84.83 ± 13.50
Corporate company	82.80 ± 12.94	83.37 ± 11.52	80.77 ± 12.36	86.02 ± 13.99	77.27 ± 13.93
Retail services	82.69 ± 10.60	81.31 ± 11.81	79.21 ± 12.02	89.26 ± 10.60	73.22 ± 12.02
Retired	81.73 ± 12.08	83.25 ± 10.13	78.94 ± 11.02	88.45 ± 10.76	79.17 ± 11.85
Self-employed	82.33 ± 11.13	82.44 ± 10.49	78.64 ± 12.03	85.42 ± 12.43	78.33 ± 14.91
Other	82.14 ± 12.24	82.02 ± 12.08	78.43 ± 13.87	86.07 ± 13.08	77.14 ± 13.750
Group comparison (*p*)	0.356	0.190	0.143	0.222	0.001

Health insurance					
Urban employees	83.33 ± 12.06	83.63 ± 10.56	79.99 ± 11.59	87.89 ± 11.75	78.50 ± 12.88
Urban residents	82.20 ± 11.82	82.72 ± 11.98	79.88 ± 12.45	88.88 ± 11.94	78.74 ± 13.97
Rural residents	76.00 ± 10.93	80.00 ± 11.77	76.11 ± 11.66	82.14 ± 11.20	75.43 ± 13.41
Group comparison (*p*)	0.003	0.159	0.177	0.012	0.397

Chronic condition					
Yes	82.58 ± 11.98	83.30 ± 11.48	80.32 ± 12.22	88.56 ± 12.19	75.87 ± 15.07
No	82.51 ± 12.23	82.95 ± 10.07	79.13 ± 11.49	89.09 ± 11.41	78.72 ± 13.49
Group comparison (*p*)	0.949	0.729	0.300	0.640	0.041

Perceived health					
Poor	81.75 ± 12.71	82.17 ± 10.91	79.90 ± 11.47	88.75 ± 12.26	78.92 ± 12.25
Fair	83.57 ± 12.18	83.25 ± 10.47	79.80 ± 11.86	87.65 ± 11.64	77.76 ± 13.43
Good	81.31 ± 11.42	83.58 ± 11.81	79.35 ± 11.93	87.11 ± 11.96	78.93 ± 13.19
Group comparison (*p*)	0.161	0.642	0.918	0.608	0.627

Distance to nearest community health center (minutes)					
≤15	82.41 ± 13.12	83.05 ± 11.14	78.68 ± 12.08	88.21 ± 11.89	77.53 ± 13.61
15–30	82.94 ± 11.68	83.17 ± 10.59	80.57 ± 11.10	89.11 ± 12.33	76.33 ± 14.20
>30	82.31 ± 12.33	83.29 ± 11.26	80.90 ± 12.53	88.93 ± 11.54	76.91 ± 15.77
Group comparison (*p*)	0.881	0.981	0.077	0.787	0.769
Preferred health provider					
Community facility	82.74 ± 12.20	83.0 ± 11.09	80.18 ± 11.69	85.69 ± 9.30	80.29 ± 13.37
Secondary hospital	81.41 ± 11.98	85.00 ± 9.22	78.97 ± 11.47	89.23 ± 12.10	77.05 ± 14.61
Tertiary hospital	82.32 ± 11.74	82.2 ± 11.18	79.24 ± 12.99	88.25 ± 11.97	76.53 ± 14.66
Group comparison (*p*)	0.809	0.592	0.702	0.012	0.001

Preferred care					
TCM	85.18 ± 12.88	86.40 ± 10.02	82.09 ± 12.53	88.75 ± 12.85	80.83 ± 12.90
Western medicine	80.40 ± 11.09	80.37 ± 11.23	76.08 ± 10.85	85.40 ± 12.05	74.59 ± 12.24
Integrated	83.46 ± 12.29	84.43 ± 10.55	82.07 ± 11.49	89.38 ± 10.81	80.80 ± 13.31
Group comparison (*p*)	0.003	0.000	0.000	0.003	0.000

First visit to the TCM unit					
Yes	79.73 ± 11.69	79.9 ± 11.35	77.20 ± 12.15	85.22 ± 12.22	73.13 ± 13.82
No	83.67 ± 12.04	84.4 ± 10.57	80.95 ± 11.73	90.16 ± 11.50	78.42 ± 14.59
Group comparison (*p*)	0.001	0.000	0.000	0.000	0.000

Visits to community health services over the past month					
<5	83.89 ± 12.18	85.46 ± 10.27	82.07 ± 11.7	89.34 ± 11.66	79.74 ± 12.52
5–9	81.68 ± 12.00	81.67 ± 11.42	78.40 ± 12.2	87.00 ± 11.56	77.95 ± 13.85
≥10	81.86 ± 11.78	82.01 ± 10.26	77.31 ± 9.00	85.09 ± 12.89	75.34 ± 11.68
Group comparison (*p*)	0.162	0.001	0.002	0.028	0.071

TCM visits community health services over the past month					
<5	84.18 ± 12.21	85.74 ± 10.10	82.22 ± 11.52	89.42 ± 11.56	80.56 ± 12.51
5–9	81.52 ± 11.94	81.36 ± 11.53	78.18 ± 12.31	86.89 ± 11.59	77.15 ± 13.83
≥10	80.81 ± 11.48	81.11 ± 9.99	76.44 ± 8.47	84.44 ± 13.02	74.88 ± 11.30
Group comparison (*p*)	0.041	0.000	0.000	0.009	0.004

Average TCM cost over the past month (¥)					
<50	83.66 ± 11.98	84.17 ± 10.74	80.70 ± 10.71	89.02 ± 11.45	79.65 ± 12.95
50–99	82.37 ± 11.95	82.20 ± 10.74	78.59 ± 12.03	86.91 ± 11.63	77.09 ± 13.08
≥100	80.26 ± 12.88	86.01 ± 12.68	83.22 ± 12.78	88.15 ± 13.96	81.74 ± 13.44
Group comparison (*p*)	0.230	0.039	0.022	0.211	0.029

TCM modalities received in the current visit					
<2	85.70 ± 11.74	84.62 ± 10.82	81.68 ± 11.81	89.08 ± 11.58	78.30 ± 13.99
2	81.48 ± 11.27	82.20 ± 10.35	78.19 ± 10.78	86.98 ± 12.36	76.56 ± 11.95
>2	80.32 ± 12.32	82.48 ± 11.46	78.82 ± 12.25	86.82 ± 11.64	79.55 ± 13.05
Group comparison (*p*)	0.000	0.100	0.020	0.152	0.150

Purpose of the current visit					
Disease treatment	81.57 ± 12.10	82.5 ± 10.67	79.06 ± 11.47	88.70 ± 11.57	76.11 ± 13.55
Preventive care	83.44 ± 11.69	83.9 ± 12.00	80.97 ± 13.26	87.67 ± 12.81	75.98 ± 16.71
Rehabilitation	84.14 ± 12.15	83.9 ± 10.83	80.93 ± 11.93	89.79 ± 11.92	79.56 ± 14.78
Group comparison (*p*)	0.121	0.416	0.236	0.442	0.086

**Table 3:** Predictors of perceived quality of care in traditional Chinese medicine (TCM)—results (standardized *β* coefficient) of linear regression models.

Variable	Tangibility		Reliability		Responsiveness		Assurance		Empathy	
	*β*	*p*	*β*	*p*	*β*	*p*	*β*	*p*	*β*	*p*
Gender										
Male (reference)										
Female	—	—	0.130	0.004	0.119	0.007	—	—	—	—

Education										
≤ Primary school (reference)										
Junior high school	—	—	0.123	0.010	—	—	—	—	—	—
Senior high school	—	—	—	—	—	—	−0.118	0.010	−0.133	0.003
University	—	—	0.192	<0.001	0.208	<0.001	—	—	—	—

Job										
Public institution (reference)										
Retail and services	—	—	—	—	—	—	0.099	0.032	−0.104	0.023

Health insurance										
Urban employees (reference)										
Urban residents	—	—	—	—	—	—	—	—	—	—
Rural residents	−0.157	<0.001	—	—	—	—	−0.128	0.004	—	—

Preferred health service										
TCM (reference)										
Western medicine	−0.129	0.004	−0.181	<0.001	−0.261	<0.001			−0.196	<0.001

First visit to the TCM unit										
Yes (reference)										
No	—	—	—	—	—	—	0.193	<0.001	-	-

Visits to community TCM										
<5 (reference)										
5–9	—	—	−0.105	0.020	—	—	—	—	—	—
≥10	—	—	—	—	—	—	−0.122	0.008	−0.097	0.028

TCM cost (¥ yuan)										
<50 (reference)										
≥100	—	—	—	—	0.108	0.013	—	—	0.104	0.018
TCM modalities received										
<2 (reference)										
= 2	−0.149	0.003	—	—	—	—	—	—	—	—
>2	−0.214	<0.001	—	—	—	—	—	—	—	—

## Data Availability

All data in this article is true and can be provided upon reasonable request.

## Abbreviations

TCM: Traditional Chinese medicine

## Supplementary Materials

Supplementary Materials Appendix: Table 1 Perceived quality of traditional Chinese medicine care in community health services questionnaire.
