# Supplementary material for: Perceived Quality of Traditional Chinese Medicine Care in Community Health Services: A Cross-Sectional Survey in Hangzhou of China
Source: Evid Based Complement Alternat Med. 2022 Jul 11;2022:7512581. doi: 10.1155/2022/7512581 (PMC9293547; doi:10.1155/2022/7512581)
Supplement: Supplementary Materials — Appendix: Table 1 Perceived quality of traditional Chinese medicine care in community health services questionnaire. [file 7512581.f1.docx]

Appendix

Table1 Perceived quality of traditional Chinese medicine care in community health services questionnaire

| Dimensions | Item |
| --- | --- |
| Tangibility | Q1 The layout of the TCM department is reasonable and the location is prominent and easy to find |
|  | Q2 Complete TCM technical facilities and a wide variety of TCM decoction pieces can meet your medical needs |
|  | Q3 The characteristic technology of traditional Chinese medicine health services is attractive and prompts you to come to see a doctor |
|  | Q4 Neat and warm clothing for medical staff |
|  | Q5 Comfortable and quiet environment in the traditional Chinese medicine diagnosis and treatment area |
| Reliability | Q6 During the diagnosis and treatment process, you will not worry about your own safety |
|  | Q7 During the diagnosis and treatment process, you will not worry about your privacy |
|  | Q8 The doctor will give you a clear diagnosis and inform you about the relevant treatment and medication methods, etc. |
|  | Q9 Your medical services are properly recorded on the medical record |
|  | Q10 Reasonable prices for the entire Chinese medicine health service |
|  | Q11 Your disease is under control and your health improves after treatment |
| Responsiveness | Q12 Agency staff are always happy to serve you |
|  | Q13 The institution discloses medical service information such as the areas of expertise of each TCM physician and the time of visiting |
|  | Q14 The entire TCM health service process is clear |
|  | Q15 Short waiting time for registration, payment, waiting, medicine collection, etc. |
| Assurance | Q16 The technical level and professional quality of TCM physicians can solve your problems |
|  | Q17 Medical staff can answer your doubts in a timely, smooth and effective manner |
|  | Q18Medical staff always maintain a rigorous, serious and enthusiastic attitude |
| Empathy | Q19 Doctors take the initiative to give you related disease science, traditional Chinese medicine psychological counseling and health education, etc. |
|  | Q20 Physicians are always "patient-focused" prioritizing your health, financial, etc. interests |
